# Agreement between activPAL and ActiGraph for assessing children's sedentary time

**DOI:** 10.1186/1479-5868-9-15

**Published:** 2012-02-19

**Authors:** Nicola D Ridgers, Jo Salmon, Kate Ridley, Eoin O'Connell, Lauren Arundell, Anna Timperio

**Affiliations:** 1 Centre for Physical Activity and Nutrition Research, Deakin University, 221 Burwood Hwy, Burwood 3125 Melbourne, Australia; 2 School of Education, Flinders University, Adelaide, Australia

**Keywords:** Accelerometry, Children, Objective assessment, Sedentary time, Sedentary behavior

## Abstract

**Background:**

Accelerometers have been used to determine the amount of time that children spend sedentary. However, as time spent sitting may be detrimental to health, research is needed to examine whether accelerometer sedentary cut-points reflect the amount of time children spend sitting. The aim of this study was to: a) examine agreement between ActiGraph (AG) cut-points for sedentary time and objectively-assessed periods of free-living sitting and sitting plus standing time using the activPAL (aP); and b) identify cut-points to determine time spent sitting and sitting plus standing.

**Methods:**

Forty-eight children (54% boys) aged 8-12 years wore a waist-mounted AG and thigh-mounted aP for two consecutive school days (9-3:30 pm). AG data were analyzed using 17 cut-points between 50-850 counts·min^-1 ^in 50 counts·min^-1 ^increments to determine sedentary time during class-time, break time and school hours. Sitting and sitting plus standing time were obtained from the aP for these periods. Limits of agreement were computed to evaluate bias between AG50 to AG850 sedentary time and sitting and sitting plus standing time. Receiver Operator Characteristic (ROC) analyses identified AG cut-points that maximized sensitivity and specificity for sitting and sitting plus standing time.

**Results:**

The smallest mean bias between aP sitting time and AG sedentary time was AG150 for class time (3.8 minutes), AG50 for break time (-0.8 minutes), and AG100 for school hours (-5.2 minutes). For sitting plus standing time, the smallest bias was observed for AG850. ROC analyses revealed an optimal cut-point of 96 counts·min^-1 ^(AUC = 0.75) for sitting time, which had acceptable sensitivity (71.7%) and specificity (67.8%). No optimal cut-point was obtained for sitting plus standing (AUC = 0.51).

**Conclusions:**

Estimates of free-living sitting time in children during school hours can be obtained using an AG cut-point of 100 counts·min^-1^. Higher sedentary cut-points may capture both sitting and standing time.

## Background

There is increasing interest in the effects of sedentary behaviors on children's and adults' health [1,2] largely due to emerging evidence that objectively-assessed sedentary time is associated with cardio-metabolic health [3-5]. The ActiGraph (AG) accelerometer has been commonly used in the objective assessment of sedentary time. However, there is considerable variability in the cut-points used to identify sedentary time using this accelerometer in child populations. AG sedentary time cut-points used in school-aged children and adolescents have included 100 counts·min^-1 ^[6,7], 200 counts·min^-1 ^[8], 500 counts·min^-1 ^[3,4], and 800 counts·min^-1 ^[9], yet only two thresholds (100 and 800 counts·min^-1^) have been validated [6,7,9].

Objective measures such as accelerometers estimate sedentary time based on a lack of movement [10]. Sedentary behavior is typically defined as sitting behaviors that require low levels of energy expenditure to perform (≤1.5 METS) [11], and a lack of movement may indicate low levels of energy expenditure when using an accelerometer. Time spent in sedentary behavior is distinct from the lack of physical activity, which is defined as the amount of time not spent engaged in physical activity of a particular intensity (often moderate-to-vigorous physical activity), and often incorporates light intensity physical activity behaviors [12]. It is possible, however that low movement may be recorded by a hip-mounted accelerometer, but the individual could be standing (which is a very light intensity activity [12]) therefore more energy may be expended than that typically associated with sedentary behaviors [1]. Though the differences in energy expenditure may be considered negligible, the accumulation of these differences may have implications for energy balance over time [13].

In recent years, opportunities for measuring patterns of sitting/lying time (referred to as sitting time hereon in) have been made possible through the use of inclinometers (e.g. activPAL [aP], PAL Technologies Ltd, Glasgow, UK) to detect postures. To date, however, no studies have used the aP to determine the accuracy of AG cut-points for assessing sitting time or to identify whether sitting can be differentiated from sitting and standing time. Whilst the AG is unable to provide postural information, sitting and sitting plus standing require little vertical acceleration. Consequently, research is needed to examine whether accelerometer sedentary cut-points reflect the amount of time children spend sitting, [12] particularly as the AG is likely to continue to be used to measure both sedentary time and physical activity intensities.

The aim of this study was to examine the agreement between AG cut-points for sedentary time and objectively-assessed sitting and sitting plus standing time in children using the aP during the school day. Class time and break time were also examined separately as class time is typically sedentary, while all children have opportunities for activity during recess and lunchtime. It was hypothesized that a lower AG cut-point would have greater agreement with aP sitting time and a higher AG cut-point would have greater agreement with aP sitting plus standing time. A secondary aim was to examine whether an accelerometer count cut-point could be used to determine time spent sitting and sitting plus standing.

## Method

### Participants

Following approval from the Deakin University Human Ethics Advisory Group (Health) and the Department of Education and Early Childhood Development, one school of low socioeconomic status located in Melbourne, Australia, was invited to participate in the study. Once informed written consent had been obtained from the school Principal, all children in Grades 3-6 (n = 255; aged 8-12 years) were invited to participate, with 56 children (32 boys, 24 girls; 22% response rate) returning informed written parental and student consent forms. Data were collected in November and December 2009 (late spring/early summer).

### Procedure

Participants wore an AG and aP simultaneously for two consecutive school days. Children in grades 3-4 (n = 20) were fitted with the aP and accelerometer at the start of the school day on day one by the research team, and were instructed to wear both monitors during all waking activities, except during water-based activities (such as swimming and bathing), until the end of the following school day (day 2). The researcher team monitored that the devices were worn across both days. The monitors were then collected and the data downloaded. The monitors were then distributed to children in Grades 5-6 (n = 28) on the same day the following week using the same procedure. Overall, six children were absent on data collection days, and did not receive the monitors. The final sample comprised 48 children (26 boys; 22 girls; mean age = 10.3 ± 1.2 years). All children received an active toy as compensation for their participation in the study.

### Measures

Each child wore a GT1M AG on their right hip using an adjustable nylon belt. The accelerometer is a small and lightweight monitor that measures vertical acceleration and deceleration of human motion. Detected accelerations are filtered, converted to a number (counts), and subsequently summed over a specified time interval (epoch), which in this study was 15 seconds. Firmware version 4.3.0 was used and the normal filter was selected. The AG is the most commonly used accelerometer in field-based research, and has been shown to have acceptable reliability and validity in pediatric populations [14].

The activPAL Professional is a small uni-axial accelerometer, worn midline on the anterior aspect of the thigh, which detects limb position using an inclinometer. The monitor was enclosed in a small pocket in an adjustable elasticized belt which was secured at the mid-anterior position on the child's thigh. Data concerning limb position are sampled at 10 Hz, and this information was used to estimate time spent sitting/lying, upright or walking in 15-second epochs [15]. While the aP has not been validated for measuring sitting time in school-age children, it has demonstrated acceptable reliability and validity for measuring sitting time in adults [16].

### Data management

Data were downloaded using aP (v5.8.3.5) and AG (v4.2.0) software and initially screened for compliance to the procedure. Two children did not return monitors to school, and were excluded from the study. To be included in the analyses, children had to have worn both monitors for two complete school days (9 am to 3:30 pm). Furthermore, since the evaluation of compliance in wearing the accelerometer is often a contentious issue in field-based research, this approach ensured that zero counts were indicative of no movement and could be retained for analyses. All children who returned the monitors met these criteria.

AG and aP data were matched by day and time and processed using a customized macro. The processed data were handled in two ways. Firstly, a number of different count thresholds were used to define sedentary time using the AG data. Total durations of counts below 50 counts·min^-1 ^(AG50) and in increments increasing by 50 counts·min^-1 ^up to 850 counts·min^-1 ^(total of 17 cut-points) were extracted to reflect the range of different cut-points used to define sedentary time in the literature to date [3,4,6-9]. Sedentary time was defined as the number of minutes that the count data were below these specified cut points. The number of minutes spent sitting, upright and stepping were obtained from the aP for each day. Seconds of stepping were subtracted from time spent upright to compute time spent standing (upright) but not stepping. On both days, time spent sedentary (AG), and sitting and sitting plus standing (aP) were computed for class time, recess and lunch time (break time) and total school hours (9 am to 3:30 pm). Data were averaged across the two days. Data recorded outside of school hours were excluded. Secondly, AG and aP epochs were also individually matched by day and time. Dichotomized variables were created to categorize each epoch as a) sitting or not sitting (aP), b) sitting plus standing or not sitting plus standing (aP), or c) sedentary or not sedentary as defined using the 17 different AG cut-points. Data were extracted for class time, break time and total school hours and used in subsequent analyses.

### Statistical analyses

Descriptive statistics were calculated for all variables. Percentage agreement between the AG and the aP (e.g. AG output at a specified cut-point classes epoch as sedentary time and aP identifies an epoch as sitting) was initially determined using the dichotomized data. The Bland-Altman method [17] was used to evaluate the bias and limits of agreement between the 17 sedentary cut-points from AG50 to AG850 and aP sitting and sitting plus standing time during class time, break time and the school day using the continuous data (min/day). Analyses were conducted in Stata 11.0, and statistical significance was set at *p *< 0.05. Concurrent time interval data (expressed as a median) across school hours were plotted to visually examine patterns of sitting and sitting plus standing against the 17 different AG cut-points.

Receiver operating characteristic (ROC) curve analyses were performed using MedCalc v.11.4.2.0 (MedCalc Software, Belgium) using the dichotomized data. ROC analysis provides an empirical basis for determining appropriate cut-points with the aim of reducing misclassification through examination of sensitivity (true positive rate) and specificity (false positive rate). The area under the curve (AUC) represents the accuracy of a cut-point, with ROC AUC values of ≥ 0.90 considered excellent, 0.80-0.89 good, 0.70-0.79 fair, and < 0.70 poor [18]. Data from 50% (n = 24) of the children were randomly selected to identify cut-points which maximized the sensitivity and specificity for sitting and sitting plus standing time. The identified cut-points were then cross-validated in the remaining children (n = 24) as previously recommended [19].

## Results

**Figure 1:**
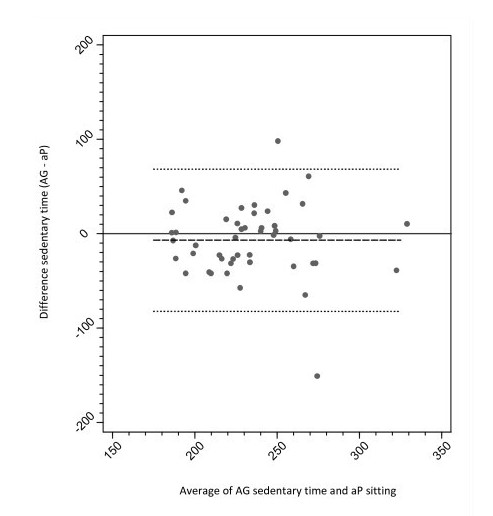
**Bland-Altman plot of the difference between time spent sedentry (ActiGraph 100 counts -min^-1^) and time spent sitting (aP)**.

The time spent sedentary according to AG cut-points and time spent sitting and sitting plus standing according to the aP is shown in Table 1. On average, the aP revealed that children spent 218.9 minutes and 315.5 minutes of the school day sitting and sitting plus standing. This equated to 56.1% and 80.9% of the school day (total duration = 390 minutes) spent sitting and sitting plus standing, respectively. According to the AG cut-points, children were sedentary for 192 minutes (AG50) to 309.7 minutes (AG850) of the school day. Table 2 presents the percentage agreement, mean differences and 95% limits of agreement using the Bland-Altman method,[17] between aP sitting time and the 17 AG thresholds between AG50 to AG850 for class time, break time and school hours. The level of agreement was moderate to high for AG50 (69-70.8%). The lowest percentage agreement was for AG850 (36-62.8%). The lowest mean bias for sitting time, regardless of direction, was AG150 for class time (3.8 minutes), AG50 for break time (-0.8 minutes), and AG100 for the school day (-5.2 minutes). However, the 95% limits of agreement were wide for these thresholds, and ranged from 49.4 minutes for break time (AG50) to 144.7 minutes for the school day (AG100). A Bland-Altman plot demonstrating the agreement between aP and AG100 for the school day is shown in Figure 1.

**Table 1:** Mean (range) time (minutes) spent sedentary according to activPAL and ActiGraph cut-points

	Class time (min) (300 min/day)	Break time (min) (90 min/day)	School Day (min) (390 min/day)
**activPAL**			

**Sitting**	189.9 (137.4, 256.6)	28.9 (7.3, 67.2)	218.9 (150.2, 321.9)

**Sitting plus standing**	257.3 (230.1, 283.4)	58.2 (35.6, 83.5)	315.5 (284.2, 364.0)

**ActiGraph (cut-point)**			

**50**	163.8 (109.9, 238.8)	28.2 (12.3, 63.6)	192.0 (129.5, 302.4)

**100^1^**	181.3 (134.6, 249.8)	32.4 (14.8, 65.9)	213.6 (157.5, 315.6)

**150**	193.7 (150.0, 256.3)	35.7 (17.4, 67.9)	229.4 (176.3, 324.1)

**200^2^**	202.7 (161.3, 261.0)	38.3 (19.8, 68.6)	241.0 (190.9, 329.6)

**250**	210.3 (172.4, 265.3)	40.7 (21.4, 70)	251.0 (203.8, 335.3)

**300**	216.2 (181.4, 268.1)	42.7 (22.2, 71.3)	258.9 (213.6, 339.4)

**350**	221.7 (189.6, 271.3)	44.7 (23.9, 71.9)	266.4 (223.6, 343.1)

**400**	226.2 (196.5, 272.6)	46.3 (25.0, 73.3)	272.4 (231.1, 345.1)

**450**	230.5 (201.3, 275.0)	47.8 (26.0, 75.3)	278.3 (238.9, 348.3)

**500^3^**	234.1 (205.4, 276.5)	49.1 (26.4, 77.5)	283.2 (245.1, 350.0)

**550**	237.6 (209.5, 277.6)	50.5 (27.9, 78.4)	288.1 (250.1, 351.6)

**600**	240.6 (213.3, 278.3)	51.7 (29.1, 79.3)	292.2 (253.5, 352.8)

**650**	243.4 (217.3, 280.1)	52.8 (29.9, 79.8)	296.3 (261.1, 354.9)

**700**	245.8 (220.1, 281.4)	53.3 (30.5, 80.0)	299.1 (265.5, 356.6)

**750**	248.3 (224.4, 282.1)	55.1 (32.5, 80.8)	303.3 (269.4, 358.1)

**800^4^**	250.5 (227.9, 282.8)	56.1 (33.8, 81.4)	306.6 (272.6, 359.0)

**850**	252.6 (229.9, 283.5)	57.1 (34.9, 81.5)	309.7 (277.3, 360.0)

^1 ^Treuth et al. [7]; Evenson et al. [6]; ^2 ^Riddoch et al. [8]; ^3 ^Ekelund et al. [3]; Sardinha et al. [4]; ^4 ^Puyau et al. [9]

**Table 2:** Concurrent comparison between sedentary time using different Actigraph (AG) cut-points and activPAL (aP) sitting time

AG Cut-point	Class time			Break time			School day		
	
	Agreement (%)	Mean Difference (AG-aP)	95% LoA	Agreement (%)	Mean Difference (AG-aP)	95% LoA	Agreement (%)	Mean Difference (AG-aP)	95% LoA
**50**	70.8	-26.1	-83.9 to 31.7	69.0	-0.8	-25.5 to 23.9	70.4	-26.9	-103.4 to 49.7

**100^1^**	70.6	-8.7	-62.6 to 45.2	64.7	3.4	-20.9 to 27.8	69.4	-5.2	-77.6 to 67.1

**150**	70.2	3.8	-47.3 to 54.8	61.4	6.8	-17.4 to 31.0	68.2	10.6	-58.9 to 80.0

**200^2^**	69.4	12.8	-36.9 to 62.5	58.4	9.3	-15.0 to 33.6	66.9	22.1	-46.0 to 90.3

**250**	68.8	20.4	-28.9 to 69.7	55.8	11.7	-12.7 to 36.2	65.8	32.1	-35.6 to 99.8

**300**	68.0	26.3	-22.6 to 75.0	53.3	13.8	-10.7 to 38.3	64.6	40.0	-27.1 to 107.1

**350**	67.4	31.7	-16.4 to 79.8	51.1	15.8	-9.1 to 40.7	63.6	47.5	-19.2 to 114.2

**400**	66.7	36.2	-12.3 to 84.7	49.0	17.3	-7.7 to 42.4	62.6	53.5	-13.6 to 120.7

**450**	66.1	40.6	-8.3 to 89.4	47.0	18.9	-6.2 to 43.9	61.7	59.4	-7.9 to 126.8

**500^3^**	65.5	44.2	-5.2 to 93.5	45.1	20.1	-4.9 to 45.2	60.8	64.3	-3.4 to 131.9

**550**	65.0	47.7	-2.2 to 97.5	43.5	21.5	-3.6 to 46.6	60.1	69.2	0.9 to 137.5

**600**	64.5	50.6	0.8 to 100.5	41.9	22.7	-2.5 to 47.9	59.3	73.3	5.0 to 141.7

**650**	64.1	53.5	3.4 to 103.6	40.5	23.9	-1.2 to 49.0	58.7	77.4	8.9 to 145.9

**700**	63.7	55.9	5.4 to 106.4	39.2	24.4	-0.6 to 49.3	58.1	80.9	12.2 to 149.6

**750**	63.4	58.3	7.5 to 109.2	38.0	26.1	1.2 to 51.0	57.5	84.5	15.4 to 153.5

**800^4^**	63.1	60.6	9.2 to 111.9	36.9	27.1	2.1 to 52.2	57.0	87.7	18.1 to 157.1

**850**	62.8	62.6	11.3 to 114.1	36.0	28.2	3.2 to 53.1	56.6	90.8	21.3 to 160.3

^1 ^Treuth et al. [7]; Evenson et al. [6]; ^2 ^Riddoch et al. [8]; ^3 ^Ekelund et al. [3]; Sardinha et al. [4]; ^4 ^Puyau et al. [9]

**Figure 2:**
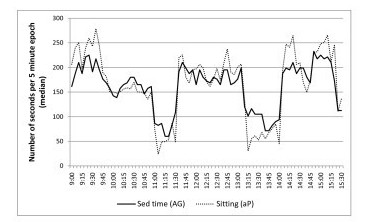
**Concurrent measurement pattern of aP sitting time and AG sedentary time defined as 100 counts min^-1^**.

**Figure 3:**
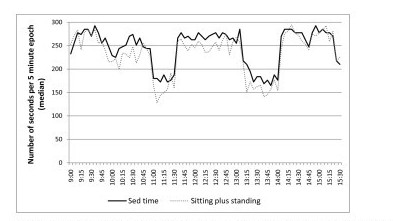
**Concurrent measurement pattern of aP sitting plus standing time and AG sedentary time defined as 850 counts min^-1^**.

Table 3 reports the percentage agreement, mean differences and 95% limits of agreement between aP sitting plus standing time and AG thresholds. The highest level of agreement for sitting plus standing time was AG250 for class time (79.5%), AG50 for break time (70.8%), and AG200 for the whole school day (76.6%). The smallest bias for sitting plus standing time was AG850 for class time (-4.7 minutes), break time (-1.1 minutes) and the school day (-5.8 minutes). The 95% limits of agreement were 38.8 minutes (class time), 28.1 minutes (break time), and 63 minutes (school day) based on the smallest mean differences across the school day. Figures 2 and 3 illustrate the concurrent measurement patterns of sitting and sitting plus standing time in 5 minute intervals across school hours for AG100 and AG850, respectively, based on the findings from the Bland-Altman analyses above.

**Table 3:** Concurrent comparison between sedentary time using different Actigraph (AG) cut-points and activPAL (aP) sitting plus standing time

AG Cut-point	Class time			Break time			School day		
	
	Agreement (%)	Mean Difference (AG-aP)	95% LoA	Agreement (%)	Mean Difference (AG-aP)	95% LoA	Agreement (%)	Mean Difference (AG-aP)	95% LoA
**50**	74.4	-93.5	-143.4 to -43.6	70.8	-30.0	-48.6 to -11.5	73.6	-30.0	-48.6 to -11.5

**100^1^**	77.4	-76.0	-119.3 to -32.8	70.1	-25.8	-43.3 to -8.4	75.7	-25.8	-43.3 to -8.4

**150**	78.8	-63.6	-101.2 to -26.0	69.0	-22.5	-39.1 to -5.8	76.5	-22.5	-39.1 to -5.8

**200^2^**	79.2	-54.6	-88.4 to -20.8	67.7	-19.9	-36.1 to -3.8	76.6	-19.9	-36.1 to -3.8

**250**	79.5	-47.0	-77.7 to -16.2	66.3	-17.5	-33.4 to -1.6	76.4	-17.5	-33.4 to -1.6

**300**	79.4	-41.1	-69.6 to -12.6	64.9	-15.5	-31.1 to 0.2	76.1	-15.5	-31.1 to 0.2

**350**	79.3	-35.6	-61.7 to -9.6	63.6	-13.5	-29.1 to 2.1	75.6	-13.5	-29.1 to 2.1

**400**	79.1	-31.1	-55.7 to -6.6	62.2	-11.9	-27.5 to 3.6	75.2	-11.9	-27.5 to 3.6

**450**	78.8	-26.8	-49.9 to -3.7	60.8	-10.4	-25.7 to 4.9	74.6	-10.4	-25.7 to 4.9

**500^3^**	78.5	-23.2	-45.5 to -0.9	59.5	-9.1	-24.2 to 6.0	74.1	-9.1	-24.2 to 6.0

**550**	78.2	-19.7	-41.3 to 2.0	58.2	-7.8	-22.9 to 7.4	73.6	-7.8	-22.9 to 7.4

**600**	78.0	-16.8	-37.8 to 4.3	57.0	-6.5	-21.4 to 8.3	73.1	-6.5	-21.4 to 8.3

**650**	77.7	-13.9	-34.2 to 6.5	55.9	-5.4	-20.0 to 9.3	72.7	-5.4	-20.0 to 9.3

**700**	77.5	-11.5	-31.5 to 8.6	54.9	-4.9	-19.4 to 9.7	72.3	-4.9	-19.4 to 9.7

**750**	77.3	-9.0	-28.9 to 10.8	54.0	-3.1	-17.3 to 11.1	71.9	-3.1	-17.3 to 11.1

**800^4^**	77.0	-6.8	-26.4 to 12.8	53.0	-2.1	-16.4 to 12.1	71.5	-2.1	-16.4 to 12.1

**850**	76.9	-4.7	-24.1 to 14.7	52.3	-1.1	-15.1 to 13.0	71.2	-1.1	-15.1 to 13.0

^1 ^Treuth et al. [7]; Evenson et al. [6]; ^2 ^Riddoch et al. [8]; ^3 ^Ekelund et al. [3]; Sardinha et al. [4]; ^4 ^Puyau et al. [9]

According to ROC analyses, the optimal sensitivity and specificity based on the AUC (0.75) for sitting time was at an accelerometer cut-point of 24 counts per 15 second epoch (96 counts·min^-1^). The sensitivity and specificity of this cut-point were 71.7% and 67.8%, respectively. In the cross-validation group, the sensitivity, specificity and percentage agreement were 71.4%, 70.8% and 71.1% respectively. For sitting plus standing time, the AUC was poor (0.51). Based on the recommendations of Welk [19], no further analyses were undertaken.

## Discussion

This is the first study to examine the agreement between AG cut-points for sedentary time and objectively-assessed periods of free-living sitting and sitting plus standing time in children using the aP, and to examine whether an accelerometer count cut-point could be used to determine time spent sitting and sitting plus standing. This study found that during school hours, the lowest mean bias (-5.2 minutes) between AG sedentary time and aP sitting time was observed for an AG cut-point of 100 counts·min^-1 ^in this age group of children. Furthermore, the ROC curve analysis for sitting time provided an optimal cut-point of 96 counts.min^-1 ^(24 counts per 15 seconds), which had reasonable agreement, sensitivity and specificity in the cross-validation group. This provides support to previous studies that have determined that 100 counts.min^-1 ^was the optimal cut-point for measuring youth sedentary time in free-living conditions [6,7], which also had an excellent ability to classify sedentary time in children [20]. Though it should be noted that the present study's sensitivity and specificity were lower than previous studies [6,20], this is the first study to use postural information as the criterion measure, demonstrating that a cut-point of 100 counts·min reflects the time children spend sitting.

It should be noted that while the mean bias suggested that 100 counts.min^-1 ^provided good agreement with aP sitting time, the limits of agreement were wide (range -77.6 to 67.1 minutes). This indicates that while the mean difference is small at a group level, the variance is larger and reflects a greater degree of under- and over-estimation at the individual level between the aP and the AG. This degree of variability at the individual level may be problematic in determining behavior change at this level following an intervention, for example. The wide limits of agreement may be attributable, to some extent, to the way that sedentary time is determined and the positioning of the monitor [21]. The aP uses a thigh-mounted inclinometer to obtain information concerning posture, whilst the hip-mounted AG determines sedentary time due to a lack of vertical displacement. Interestingly, while these monitors are measuring different outcome variables, which mean that a discrepancy will occur between the monitor's outputs, the group average concurrent measurement pattern between the AG and the aP depicted in Figure 1 was similar at 100 counts.min^-1^.

Several studies have examined the utility of the AG to detect sedentary time in adults. Hart et al. [22] found that an AG cut-point of 50 counts.min^-1 ^may be a better estimate of sitting time (when using the 7164 model). The present study found the highest percentage agreement between sitting and an AG cut-point of 50 counts.min^-1^, which somewhat supports this finding. Estimates of the validity of the 100 counts.min^-1 ^cut-point in adults has been mixed, with this threshold resulting in significantly more sedentary time when using the AG GT1M model compared to the aP [21], whilst others found it underestimated sedentary time [23]. In the latter study, a cut-point of 150 counts.min^-1 ^was the most accurate threshold for defining sitting time using the aP as the criterion, which is consistent with the finding for class time in the present study. It should be noted, however, that a GT3X with the low frequency extension filter option selected was used [23], which may account for some of the variability observed between studies. This option extends the lower threshold for signal detection, as it was found that a higher level of acceleration was needed to generate counts in the GT1M and GT3X AG models compared to the 7164 [24].

There is wide variation in published AG cut-points used to define sedentary time in children [25]. In the present study, a smaller mean bias was observed for AG500 [3,4] and AG800 [9] for sitting plus standing time, compared to sitting time. Previous studies have found that higher cut-points in adults detected more sedentary time compared with time spent sitting from the aP [21,23]. Trost and colleagues [20] found of the commonly used sedentary cut-points, AG800 had fair classification accuracy and low specificity, indicating that this cut-point was incorrectly classifying activity as sedentary time [19]. Overall, the findings from the present study and previous studies suggest that higher AG cut-points are capturing more activity than can be associated with sitting time, therefore studies that have used higher AG sedentary cut-points should be viewed with this limitation in mind. A limitation of hip-mounted accelerometers is their susceptibility of misclassifying standing light-to-moderate intensity activities as sedentary [20,26]. In the present study the ROC curve analyses for sitting plus standing resulted in a poor AUC, which meant that the associated cut-point would be ineffective characterizing sitting plus standing. This demonstrates that the AG cannot differentiate sitting from standing with minimal movement, and that researchers interested in examining time spent sitting plus standing should use objective monitors with inclinometers, such as the aP [23].

This study found that agreement between aP and the AG derived sedentary time varied depending on the period of day that was being examined. The lowest mean bias for break time and class time were observed at AG50 and AG150, respectively, for sitting time though the limits of agreement were also wide at these thresholds. At a practical level, it is unlikely that different cut-points are needed to assess sedentary time during different parts of the day. However, it appears that these findings reflect the variability in children's sitting time across the day. For example, sitting accounts for a small proportion of break time [27], yet accounts for a large proportion of class time [25]. Future studies that aim to reduce time spent sitting during specific periods of school hours should be aware of such bias when assessing the effectiveness of different strategies.

There are several limitations that warrant attention. Firstly, no true criterion of sedentary time, such as direct observation, was used in this study. While the aP has been validated for assessing sitting time in preschool [28] and adult populations [16], it has not yet been validated in school-age child populations. Secondly, the monitor used in this study was the GT1M, which has been succeeded by the GT3X and the GT3X+ AG models. While there are emerging data that the activity counts are comparable between the GT1M and the GT3X in adults [29], differences have been noted in low count ranges [24]. As such, these findings are only generalizable to data collected using the normal filter. Thirdly, data analyses were restricted to two school days (9 am-3:30 pm), as children wore both monitors simultaneously for two days during this time only. Further research should examine the agreement between the aP and the AG during waking hours across multiple days. It should be noted, however, that 100% compliance during the school day meant that consecutive zeros were indicative of no movement rather than non-wear, which is a strength of this study.

## Conclusion

An AG cut-point of 100 counts·min^-1 ^provided a good estimate of free-living sitting time in children during school hours. Higher cut-points that have been used to report children's sedentary time may capture both sitting and standing time. Further research is needed to examine the use of the 100 counts·min^-1 ^cut-point to determine sitting time across the whole day, and against health indices in children.

## Abbreviations

AG: ActiGraph; aP: activPAL; AUC: Area Under the Curve.

## Competing interests

The authors declare that they have no competing interests.

## Authors' contributions

JS conceived the study and secured the funding. JS and LA planned the study design. LA collected the data. EOC performed the initial data manipulation. NDR performed the statistical analyses. NDR, JS, AT and KR and interpreted the data. NDR wrote the manuscript. AT, KR, JS, LA and EOC critically reviewed and revised the manuscript. All authors read and approved the final version of the manuscript.
